# Is there an increased risk of preeclampsia using donor sperm, depending on sexual condition?

**DOI:** 10.5935/1518-0557.20250155

**Published:** 2026

**Authors:** Begoña Prieto, Natalia Sanz, María Díaz-Nuñez, Lucía Lainz, Silvia Pérez-Fernández, Olatz Molina, Ainara Bengoetxea, Maitane Gantxegi, Roberto Matorras

**Affiliations:** 1 Human Reproduction Unit, Cruces University Hospital, Baracaldo, Spain; 2 Instituto Valenciano de Infertilidad (IVI), IVIRMA, Bilbao, Spain; 3 Obstetrics and Gynecology Department, Cruces University Hospital, Baracaldo, Spain; 4 Innovation in Assisted Reproduction Department, Biobizkaia Health Research Institute, Baracaldo, Spain; 5 Bioinformatics, Biostatistics and Information Systems Department, Biobizkaia Health Research Institute, Baracaldo, Spain

**Keywords:** insemination with donor sperm, lesbians, transgender, single women, heterosexual women, preeclampsia

## Abstract

**Objective:**

An increased risk of preeclampsia has been described when performing donor intrauterine insemination. One of the possible causes is immunological tolerance to semen. Both lesbian and single women, who have theoretically lower exposure to seminal fluid than heterosexual women requesting artificial insemination with donor, sperm should have a higher incidence of preeclampsia, although this fact has not been analyzed previously.

**Methods:**

The population under study consisted of 439 gestations <24 weeks, achieved with artificial insemination with donor sperm, performed in two different populations: heterosexual couples and SLTG (single women, lesbian women, women with transgender partner). Preeclampsia rates and other perinatal outcomes were compared.

**Results:**

No significant differences were found in the development of preeclampsia in the SLTG group 6.03% (12/199) *vs*. heterosexual patients 5.83% (14/240), *p*=0.93. In the SLTG group, compared with heterosexual group, age (36 *vs*. 35, *p*=0.002) and BMI (24.8 *vs*. 23.6, *p*=0.002) were somewhat higher. After adjusting for BMI, age and multiplicity of gestation, there were no significant differences in the risk of preeclampsia between SLTG group and heterosexual women. As expected, in women with preeclampsia, gestational ages and newborn weights (quantitative markers of preeclampsia severity) were significantly lower than in women without preeclampsia.

**Conclusions:**

Our study suggests that neither sexual orientation nor previous sperm exposure carries a higher risk of preeclampsia, especially after correction for age and weight. Artificial insemination with donor sperm performed on single women, lesbians or women with transgender partners is a safe, simple and efficient technique, just as in heterosexual couples.

## INTRODUCTION

In Europe 50,000 donor insemination cycles were performed in 2018, with Spain the country that performed the most artificial insemination with donor sperm cycles, followed by Belgium, Denmark and United Kingdom (European IVF Monitoring Consortium, 2022). Artificial insemination with donor sperm performed in Spain has increased over the last few years (Martínez-Granados *et al.*, 2022), which could be related to more permissive laws in Spain, as well as changes in family models, women with desires for single motherhood or same sex female couples, with the indication of severe male factor in heterosexual couples remaining stable.

Currently, there is a delay in the age of gestation in all family types, which seems to be more noticeable in single women (Ferrara *et al.*, 2000; Soares *et al.*, 2019). This increase in age leads to an escalation in the risk of developing higher morbidity and mortality during pregnancy, in addition to the risks associated with artificial insemination with donor sperm itself, such as hypertensive disorders (González-Comadran *et al.*, 2014; Allen *et al.*, 2021; Pohjonen *et al.*, 2022).

In the general population, a risk of developing preeclampsia has been described in industrialized countries in around 3-5% of pregnancies (Dahlstrøm *et al.*, 2006; Wallis *et al.*, 2008; Robillard *et al.*, 2011). Several meta-analyses have been carried out during the last decade (González-Comadran *et al.*, 2014; Allen *et al.*, 2021; Pohjonen *et al.*, 2022) and they have found an increased risk of preeclampsia with the use of donor insemination, compared to insemination with husband’s sperm (OR: 1.63, 95% CI 1.36-1.95) (González-Comadran *et al.*, 2014), OR: 1.77, 95 % CI 1.26-2.48 (Pohjonen *et al.*, 2022) and OR: 1.49 (1.05-2.09) (Allen *et al.*, 2021).

The pathogenesis of preeclampsia remains unclear, although there are multiple theories (Jung *et al.*, 2022), one of which is systemic vascular endothelial alteration characterized by an alteration in vascular hemostasis (Lapaire *et al.*, 2010), and another the immunological consequence of a maternal immune response to foreign fetal antigens. These antigens would be found in sperm and seminal fluid, so that some authors have pointed out that repeated exposure to paternal antigens from seminal fluid (Einarsson *et al.*, 2003; Kho *et al.*, 2009; Robillard *et al.*, 2011; Saftlas *et al.*, 2014), as well as a history of a high number of sexual cohabitations, seems to reduce the incidence of this disease (Ferrara *et al.*, 2000; Di Mascio *et al.*, 2020; Hendin *et al.*, 2022). However, other studies have not shown that exposure to semen is associated with an increased risk of preeclampsia (Cirillo *et al.*, 2022; Keukens *et al.*, 2022).

This is well characterized in preeclampsia, where limited exposure to seminal fluid, either by first conception, short period of sexual cohabitation, or use of barrier contraceptives increases risk (Klonoff-Cohen *et al.*, 1989; Dekker *et al.*, 1998). The protective effect of seminal fluid exposure is partner specific as multiparous mothers who conceive with a new partner have an increased risk of preeclampsia (Robillard *et al.*, 1993; 1994).

Furthermore, exposure to seminal fluid may have favorable consequences for fertility and gestational progression (Robertson & Sharkey, 2016). It has even been suggested that the absence of seminal fluid, which primes the immune response, may be a factor limiting endometrial receptivity and implantation success rates (Crawford *et al.*, 2015).

According to this immunological theory, lesbian women and single women, who are less exposed to seminal fluid than heterosexual women in couples requesting artificial insemination with donor sperm, should have greater rates of preeclampsia, although this fact has not been analyzed previously. In this study, we intend to evaluate whether there are differences in the development of preeclampsia in patients undergoing artificial insemination with donor sperm according to sexual status.

## MATERIAL AND METHODS

The study population consisted of all patients for artificial insemination with donor sperm between 2005 and 2019 whose cycle ended in a newborn >24 weeks. A total of 439 deliveries were counted, all of whom were attended in the same Obstetrics Department. All patients signed an informed consent. Approval for all procedures was obtained from the institutional review board on November 24, 2020, CEIC Number E20/45.

All patients underwent ovarian stimulation in the same Human Reproduction Unit. Inclusion criteria were: body mass index <35 and age <40 years, good ovarian reserve, and at least one patent tube. Adequate ovarian reserve was defined as antral follicle count ≥6 and i) in the period before 2014, FSH <12 mIUI/mL, or ii) in the period after 2015 as AMH >0.4 ng/mL in >38 years (and in <38, AMH >0.1). In heterosexual couples, the indications for AID were: azoospermia (96%), cryptozoospermia (4%).

Our artificial insemination with donor sperm procedure has been described previously (Prieto *et al.*, 2022). It consisted of the following phases. Ovarian stimulation was performed with hMG (Menopur; Ferring, Spain) or recombinant FSH (Gonal; Merck, Spain or Bemfola; Gedeon, Spain) (Prieto *et al.*, 2022). Ovarian stimulation was monitored with plasma estradiol, progesterone and ultrasound (Prieto *et al.*, 2022).

The starting dose of gonadotropins was established according to AMH, antral follicle count, woman’s age, and BMI as reported previously (Prieto *et al.*, 2022). When one or two follicles reached 18.5mm, 250mg of recombinant hCG were administered. A single insemination intrauterine insemination with 0.3 to 0.5mL was performed per cycle 38 hours after the administration of 250 mcg of recombinant hCG (Ovitrelle, Merck Laboratories, Spain). The intrauterine insemination was done via a polyethylene catheter (Frydman catheter; Imesa, Barcelona, Spain) (Matorras *et al.*, 1996). A total of six cycles of artificial insemination with donor sperm were performed if pregnancy was not obtained earlier. Donor sperm was used in every trial and the donor was changed between every artificial insemination round. Sperm donors were changed at any insemination to maximize pregnancy rates, avoiding hypothetical immunological problems. In heterosexual couples the sperm of their husbands was never used. The luteal phase was supplemented with vaginal micronized progesterone (200mg/12h for the 14 days following insemination. Donor semen samples were thawed and prepared with conventional swim-up (Origio®, Universal IVF Medium with Phenol Red).

At the first visit, patients were asked about their partner status and/or sexual orientation. Women were classified as a) Heterosexual women with male partner (n=240) b) Single heterosexual women (n=127), c) Lesbian (single or in a couple) women (n=69), d) Women with a transgender partner (n=3). No data were available on the number of previous male partners or previous use of barrier methods of contraception in any of the categories considered, except for couples with a transgender partner (none of the 3 had had a previous male partner).

### Main characteristics of the population

We studied 439 gestations achieved with artificial insemination with donor sperm, 26 of whom developed preeclampsia. 368 were singletons and 71 were twins, and 5.16% (19/368) of singletons and 9.86% (7/71) of twins developed preeclampsia (Table 1).

**Table 1:** Descriptive table of preeclampsia *vs*. no preeclampsia in the different groups.

	All	Preeclampsia	No preeclampsia	p-value
**N**	439	5.95% (26/439)	94.08% (413/439)	
**Age (years)**	36.0 [33.0;38.0]	36.0 [34.2;38.8]	35.5 [33.0;38.0]	0.453
**BMI (kg/m^2^)**	24.2 [21.7;27.9]	25.7 [24.0;28.9]	24.0 [21.6;27.8]	0.089
**Singletons (%)**	83.82 (368/439)	5.16 (19/368)	94.83 (349/368)	<0.0001
**Twin (%)**	16.17 (71/439)	9.86 (7/71)	15.50 (64/413)	0.2193
**Heterosexual couples (%)**	54.67 (240/439)	53.85 (14/26)	54.72 (226/413)	0.9307
**Single women, lesbian, women with transgender partner (%)**	45.33 (199/439)	46.15 (12/26)	45.28 (187/413)	0.9307
**-Single women (%)**	28.93 (127/439)	6.30 (8/127)	93.70 (119/127)	<0.0001
**-Lesbian (%)**	15.72 (69/439)	5.80 (4/69)	94.20 (65/69)	<0.0001
**-Women with transgender partner (%)**	0.68 (3/439)	0.00 (0/3)	100.00 (3/3)	0.4377
**Heterosexual (newborn weight, g)**	3,060 [2,685;3,395]	2,500 [1,490;2,960]	3,100 [2,700;3,430]	0.005
**Single women, lesbian, women with transgender partner (newborn weight, g)**	3,115 [2,718;3,392]	2,465 [1,890;3,092]	3,128 [2758;3,415]	0.004

The mean age of the patients was 36 years, and mean BMI was 24.8 with no significant differences between the patients who had preeclampsia and those who did not (Table 1). 45.33% (199/439) of the patients were single women, lesbians and women with a transgender partner, with the distribution being singles 28.93% (127/439), lesbians 15.72% (69/439) and transgender couples 0.68% (3/439); 54.67% (240/439) were heterosexual couples (Table 1).

The mean weight of the children born to heterosexuals who developed preeclampsia was 2,500, compared with 3,100 who did not develop preeclampsia (*p*=0.005). The mean weight of children born to the single women, lesbian and women with transgender partner group was 2,465 with preeclampsia *vs*. 3,128 without preeclampsia (*p*=0.004) (Table 1).

The definition of preeclampsia was considered as the presence of 140/90 mm Hg measured on at least two occasions at least 4-6h apart, and when proteinuria was also detected at ≥300 mg/24 hours after 20 weeks of pregnancy. We studied all gestations (simple and multiple pregnancies) with a delivery result >24 weeks, after the use of donor sperm. All deliveries were in our hospital, so the reported data could be reviewed. Preterm delivery was defined as a birth that takes place before 37 completed weeks (Zegers-Hochschild *et al.*, 2017), low birth was defined as a newborn weight under 2500g, and extreme low birth, under 1000g (Zegers-Hochschild *et al.*, 2017). Data were obtained by manual revision of clinical charts by the two same researchers (BP and NS).

### Statistical analysis

Continuous variables were tested for normality using the Shapiro-Wilks test. Variables that followed a normal distribution were presented with mean and standard deviation, whereas variables that did not follow a normal distribution were presented with a median and interquartile range. Comparison between groups was performed using Student’s t-test or the Mann-Whitney U test according to the distributional characteristics of the variable. Categorical variables were presented as frequency and percentage. Comparisons between groups were performed using the Chi-square test.

Logistic regression models were carried out to predict preeclampsia and cesarean section, adjusted for age, weight and type of couple.

All analyses were performed using R statistical software (version 4.3.1): A Language and Environment for Statistical Computing. R Foundation for Statistical Computing, Vienna, Austria.

## RESULTS

### Comparison between the single women, lesbian and women with transgender partner group and heterosexual women

We grouped patients according to the existence of seminal exposure or not, in single/lesbian/transgender *vs*. heterosexual women. With regard to clinical characteristics, age and BMI were somewhat higher in the single women, lesbian and women with transgender partner group than in heterosexual women (36 *vs*. 35 years, *p*=0.002; 24.8 *vs*. 23.6 kg/m^2^, *p*=0.002) (Table 2).

**Table 2:** Live birth rate and preeclampsia in single women, lesbian, women with transgender partner *vs*. heterosexual couples.

	Single women, lesbian, women with transgender partner	Heterosexual couples	p-value
**n**	199	240	
**Age (years)**	36.0 [34.0;38.0]	35.0 [32.0;37.0]	0.002
**BMI (kg/m^2^)**	24.8 [22.0;29.0]	23.6 [21.5;26.3]	0.002
**Pregnancy rate (%)**	26.62 (270/1015)	31.42 (339/1080)	0.016
**Live birth rate (%)**	19.60 (199/1015)	22.04 (238/1080)	0.071
**Twin (%)**	11.56 (23/199)	20.00 (48/240)	0.01
**Preeclampsia total (%)**	6.03 (12/199)	5.83 (14/240)	0.93
**Preeclampsia/single (%)**	4.54 (8/176)	5.70 (11/193)	0.61
**Preeclampsia/twin (%)**	17.39 (4/23)	6.25 (3/48)	0.15
**Perinatal Mortality (‰)**	5.03 (1/199)	16.67 (4/240)	0.30

The pregnancy rates the in single women, lesbian and women with transgender partner group was 26.62% (270/1015) *vs*. 31.42% (339/1080) in heterosexual women significantly higher (*p*=0.016), but not the live birth rate 19.60% (199/1015) *vs*. 22.04% (238/1080) (*p*=0.071) (Table 2).

The rate of preeclampsia in heterosexual couples was 5.83% (14/240), in singles 6.30% (8/127), in lesbians 5.80% (4/69), and in transgender couples 0% (0/3) (Table 1). There was no significant difference in the development of preeclampsia in the single women, lesbian and women with transgender partner group 6.03% (12/199) *vs*. heterosexual women 5.83% (14/240), *p*=0.93. The rate of preeclampsia in singleton gestations was similar in the single women, lesbian and women with transgender partner group and heterosexual women 4.54% (8/176) *vs*. 5.70% (11/193), *p*=0.61. The risk of preeclampsia in twin gestations was somewhat higher in the single women, lesbian and women with transgender partner group *vs*. heterosexual women 17.39% (4/23) *vs*. 6.25% (3/48), *p*=0.15), but without statistical significance (Table 2).

Both gestational age at delivery and newborn weight were very similar in the single women, lesbian and women with transgender partner group and heterosexual group in both single and twin gestations (Table 3).

**Table 3:** Gestational age, birthweight and cesarean section in single women, lesbian, women with transgender partner *vs*. heterosexual couples in preeclampsia and no preeclampsia.

	Single women, lesbian, women with transgender partner				Heterosexual couples				p-value
	All	Preeclampsia	No preeclampsia	p-value	All	Preeclampsia	No preeclamsia	p-value	
**Gestational age total**	39.0 [38.0;40.0]	37.0 [35.8;39.0]	39.0 [38.0;40.0]	0.011	39.0 [38.0;40.0]	36.5 [33.5;39.8]	39.0 [38.0;40.0]	0.021	0.511
**Gestational age/ single**	40.0 [38.0;40.0]	38.5 [37.0;39.2]	40.0 [38.0;40.0]	0.115	40.0 [38.0;40.0]	38.0 [34.5;40.0]	40.0 [38.0;40.2]	0.038	0.448
**Gestational age/ twin**	36.0 [35.0;37.0]	35.5 [33.8;36.2]	36.5 [35.0;37.8]	0.367	36.0 [33.5;38.0]	35.0 [32.0;35.5]	36.0 [33.8;38.0]	0.211	0.775
**Birthweight total (g)**	3120 [2720;3390]	2465 [1890;3092]	3128 [2758;3415]	0.004	3060 [2690;3390]	2500 [1490;2960]	3100 [2700;3430]	0.005	0.466
**Birthweight /single (g)**	3195 [2828;3440]	2875 [2525;3285]	3200 [2850;3450]	0.080	3170 [2865;3515]	2685 [1712;2950]	3190 [2880;3540]	0.003	0.805
**Birthweight /twin (g)**	2250 [1800;2525]	1860 [1632;2008]	2270 [1820;2580]	0.144	2375 [1672;2642]	2430 [1680;2695]	2350 [1700;2640]	0.944	0.653
**Cesarean section (%)**	23.62 (47/199)	41.67 (5/12)	22.45 (42/187)	0.37	15.83 (38/240)	64.28 (9/14)	12.95 (29/224)	0.0052	0.041
**Cesarean single (%)**	20.45 (36/176)	25.00 (2/8)	20.24 (34/168)	0.82	13.09 (25/191)	54.55 (6/11)	10.56 (19/180)	0.001	0.079
**Cesarean twin (%)**	47 (11/23)	75.00 (3/4)	42.11 (8/19)	0.64	27.66 (13/47)	100.00 (3/3)	22.73 (10/44)	0.057	0.161

There was a higher cesarean section rate in the single women, lesbian, women with transgender partner group *vs*. heterosexual group 23.62% (47/199) *vs*. 15.83% (38/240), *p*=0.04 (Table 3).

Perinatal mortality in the single women, lesbian and women with transgender partner group vs. heterosexual was 5.03‰ *vs*. 16.67‰ (*p*=0.30) (Table 2). Perinatal mortality in single pregnancies was 5.68‰ (1/176) in the single women, lesbian and women with transgender partner group *vs*. 0% (0/193) in heterosexual women (*p*=0.46). Perinatal mortality in twins was 0%(0/46) in the single women, lesbian and women with transgender partner group *vs*. 4.26% (4/94) in heterosexual women (*p*=0.30).

In the single women, lesbian, women with transgender partner group, we found a single case of mortality at 28 weeks with no data of preeclampsia. In heterosexual women, all mortalities happened in twin pregnancies. One mortality happened in the pregnancies with 1 preeclampsia (mortality at 29 weeks) and the three others in non-preeclampsia pregnancies: one mortality at 24 weeks associated with a previous feticide at 20 weeks, another associated to suspected malformation at 28 weeks, and another associated to intrauterine growth restriction at 32 weeks.

### Comparison of pregnancies with preeclampsia and pregnancies without preeclampsia

There were no significant differences in age or BMI between patients who developed preeclampsia and those who did not. Twin gestations accounted for 26.92% (7/26) of preeclampsia *vs*. 15.50% (64/413) of non-preeclampsia (*p*=0.221).

Newborn weight was significantly lower in the preeclampsia group than in the non-preeclampsia group, both in the single women, lesbian, women with transgender partner group (2,465 *vs*. 3,128, *p*=0.004) and in heterosexual women (2,500 *vs*. 3,100, *p*=0.005). The same differences were observed when singles were analyzed separately (Table 3).

Similarly, gestational age in preeclampsia was significantly lower both in the single women, lesbian and women with transgender partner group (37 *vs*. 39, *p*=0.01) and in heterosexual women (36.5 *vs*. 39, *p*=0.021) (Table 3).

When the risk of preeclampsia was adjusted for BMI, age and multiplicity of gestation, there was no significant difference between the single women, lesbian and women with transgender partner group and heterosexual women (Table 4).

**Table 4:** Preeclampsia rate adjusted by age and weight in heterosexual couples and single women, lesbian, women with transgender partner.

Preeclampsia rate adjusted by age and weight	Characteristics	OR	95% CI	*p*-value
Total gestations	Heterosexual couples Single women, lesbian, women with transgender partner Age BMI	- 1.00 1.02 1.01	- 0.43; 2.27 0.91; 1.16 0.98; 1.04	0.9 0.7 0.6
Single	Heterosexual couples Single women, lesbian, women with transgender partner Age BMI	- 0.83 1.01 0.99	- 0.30; 2.16 0.89; 1.17 0.95; 1.03	0.7 0.9 0.8
Twins	Heterosexual couples Single women, lesbian, women with transgender partner Age BMI	- 2.68 1.08 1.02	- 0.51; 15.20 0.87; 1.43 0.97; 1.08	0.2 0.5 0.4

1 OR = Odds Ratio, CI = Confidence Interval.

The cesarean section rate in preeclampsia patients was 41.67% (5/12) in the single women, lesbian and women with transgender partner group and 64.28% (9/14) in heterosexual patients (*p*=0.04), while in non-preeclampsia it was 22.45% (42/187) the single women, lesbian and women with transgender partner group and 12.95% (29/224) in heterosexual patients (*p*=0.04) (Table 3).

When the risk of cesarean section was adjusted for age, BMI and presence or absence of preeclampsia, we did not find a higher risk of cesarean section comparing the single women, lesbian and women with transgender partner group to heterosexual group (Table 5).

**Table 5:** Cesarean rate adjusted by age and weight in heterosexual couples and single women, lesbian, women with transgender partner.

Cesarean rate adjusted by age and weight	Characteristics	OR	95% CI	*p*-value
Total gestations	Preeclampsia: yes no Heterosexual couples Single women, lesbian, women with transgender partner Age BMI	5.56 - - 1.5 1.07 1.01	2.43; 13.00 - - 0.90; 2.50 0.99; 1.16 0.99; 1.03	<0.001 - - 0.12 0.10 0.2
Single	Preeclampsia: yes no Heterosexual couples Single women, lesbian, women with transgender partner Age BMI	4.37 - - 1.55 1.14 1.01	1.57; 11.8 - - 0.86; 2.82 1.03; 1.26 0.99; 1.03	0.004 - - 0.2 0.011 0.4
Twins	Preeclampsia: yes no Heterosexual couples Single women, lesbian, women with transgender partner Age BMI	14.6 - - 2.12 0.95 0.99	2.15; 295 - - 0.68; 6.59 0.81; 1.11 0.95; 1.03	0.019 - - 0.2 0.5 0.6

### Preeclampsia and insemination cycle number

We studied the development of preeclampsia/pregnancy obtained depending on the number of inseminations in which pregnancy was achieved in all groups. There were no significant differences in the development of preeclampsia between the single women, lesbian and women with transgender partner group and heterosexual women per number of inseminations (Table 6).

**Table 6:** Preeclampsia rate in single women, lesbian, women with transgender partner and heterosexual couples per performed insemination cycle.

	Preeclampsia and gestational cycle number (^*^)	Preeclampsia Single women, lesbian, women with transgender partner	Preeclampsia Heterosexual couples	p-value (95% CI)
**1^st^ cycle (%)**	5.83 (8/136)	7.54 (4/53)	4.81 (4/83)	0.33 (0.48-8.53)
**2^nd^ cycle (%)**	7.53 (11/145)	3.84 (3/78)	11.94 (8/67)	0.08 (0.08-1.16)
**3^rd^ cycle (%)**	4.34 (3/69)	6.66 (2/30)	2.56 (1/39)	0.42 (0.23-31.44)
**4^th^ cycle (%)**	4.76 (2/42)	11.1 (2/18)	0.00 (0/24)	0.20 (0.33-164.79)
**5^th^ cycle (%)**	7.14 (2/28)	7.14 (1/14)	7.14 (1/14)	1.00 (0.06-17.75)
**6^th^ cycle (%)**	0.00 (0/10)	0.00 (0/2)	0.00 (0/8)	
**7^th^ cycle (%)**	0.00 (0/2)	0.00 (0/1)	0.00 (0/1)	

(^*^) In 7 cases the conceptional cycle was not reported, none of them developing preeclampsia.

## DISCUSSION

The development of preeclampsia during pregnancy is associated with a significant increase in maternal and perinatal mortality and morbidity (ACOG - American College of Obstetricians and Gynecologists, 2020; Aracil Moreno *et al.*, 2021).

Numerous risk factors have been described, including primiparity, age, weight, twins, assisted reproductive technology, previous conditions such as diabetes, autoimmune diseases such as lupus, antiphospholipid syndrome, endocrine and fetal disorders such as hydatidiform mole (Ives *et al.*, 2020; Sanapo *et al.*, 2020; Jung *et al.*, 2022). Its etiology is not well known and numerous theories about its genesis have been postulated. One of them gives great importance to the immunological factor. In this regard, a higher rate of preeclampsia has been described in egg donation gestations (Keukens *et al.*, 2022), double gamete donation (Augusto & Margarida Póvoa, 2022) and embryo donation (Peigné *et al.*, 2023). It seems that there is immune tolerance to seminal exposure, reducing the risk of preeclampsia with increasing vaginal exposure to paternal semen over time (Einarsson *et al.*, 2003; Kho *et al.*, 2009; Robillard *et al.*, 2011; Saftlas *et al.*, 2014; Di Mascio *et al.*, 2020; Zhu *et al.*, 2021). It has been said that the duration of prior sexual cohabitation with the conceiving male partner progressively protects against pregnancy disorders (Robertson & Sharkey, 2016), a history of a high number of sexual cohabitations reduces the risk of preeclampsia (Markert, 2005) and that the use of barrier methods of contraception increases the risk of preeclampsia (Klonoff-Cohen *et al.*, 1989). On the other hand, it has been reported that the protective effect of seminal fluid exposure is partner specific as multiparous mothers who conceive with a new partner have an increased risk of preeclampsia, suggesting that the protective effect of sperm exposure is partner specific (Robillard *et al.*, 1993; 1994). However, other studies find no relationship with seminal exposure and development of preeclampsia (Hall *et al.*, 2001), while others find a relationship for artificial insemination with donor sperm, but not for IVF with donor sperm, blaming it on the higher maternal age in artificial insemination with donor sperm (Pohjonen *et al.*, 2022). Thus, the studies are confusing, and their limitations include the problem of the control group, the difficulty of making an accurate diagnosis of preeclampsia, age factor, multiparity and the fact that ART in itself increases the risk of preeclampsia.

Regarding insemination success rates, in our series the per cycle pregnancy were significantly higher among the single women, lesbian and women with transgender partner group than in heterosexual women, but not the live birth rate line with what has been described previously (Soares *et al.*, 2019; Linara-Demakakou *et al.*, 2020; Wrande *et al.*, 2022).

In our study, the risk of preeclampsia in artificial insemination with donor sperm was 5.90%, 5.14% in singletons and 9.85% in twins. In our general population of the Basque Country, the risk of preeclampsia in singleton gestations has been described as 0.73% of the total (Melchor *et al.*, 2018), at the lower limit of that described in the literature. The rate of preeclampsia in our study was much higher than generally reported in the Basque population. In agreement with this, a number of reports have shown that artificial insemination with donors sperm is associated with an increased preeclampsia risk (González-Comadran *et al.*, 2014), although other factors should be considered, such as increased age.

In the last published meta-analysis, the risk of developing preeclampsia in singleton gestations in natural conception is 2% and severe preeclampsia 0.5% (Keukens *et al.*, 2022). It is well known that assisted reproductive technology per se entails an increased risk of preeclampsia. In the aforementioned meta-analysis, the risk of preeclampsia in singletons in IVF was 4.1% and in oocyte donation 10.7% (Keukens *et al.*, 2022). The reported rates of preeclampsia in artificial insemination with donor sperm pregnancies in the literature range between 7%-10.9% in singletons and 20% in twins (Kyrou *et al.*, 2010; Fishel Bartal *et al.*, 2019). Our data on preeclampsia are lower than those described in the literature, perhaps due to a more accurate diagnosis of preeclampsia, although it should not be forgotten that also in the general population the rate of preeclampsia was lower than usually reported (Melchor *et al.*, 2018).

As mentioned, artificial insemination with donor sperm per se carries a significant risk of preeclampsia. Artificial insemination with donor sperm is a relatively frequent reproductive option in the single women, lesbian and women with transgender partner group. Given that it has been speculated that due to limited previous seminal exposure these women may be at increased risk of preeclampsia, the study of this aspect is of particular interest to avoid generating new morbidity associated with sexual orientation.

In our study, the incidence of preeclampsia was very similar in the single women, lesbian and women with transgender partner group (6.03%) and in heterosexual couples (5.83%). There were also no differences in the weights of the newborns, both the single women, lesbian and women with transgender partner group and heterosexual, nor in their gestational age, which is an indirect indicator that the severity of PE was similar in both groups.

Despite the absence of perinatal differences, it should be noted that the single women, lesbian and women with transgender partner group patients had a slightly higher mean age, with statistical significance, similar to that described by other authors (Leiblum *et al.*, 1995; Gerkowicz *et al.*, 2018; Soares *et al.*, 2019; Linara-Demakakou *et al.*, 2020), and attributable to the single women where many women mostly postpone motherhood while waiting to find a male partner (Ferrara *et al.*, 2000; Wrande *et al.*, 2022). In our population the mean weight was also higher. After multivariate analysis adjusted for age and weight, there were still no significant differences between the two groups in terms of risk of preeclampsia and rate of cesarean section. The diagnosis of preeclampsia may involve a certain degree of clinical variability, making it difficult to compare the results of the different series. Presumably, depending on the criteria used, the frequency of complications reported differs considerably. We would like to emphasize that in our study the newborns of patients with preeclampsia had a lower weight and gestational age than those of patients without preeclampsia. In our opinion, this is a good indicator of the severity of preeclampsia.

We have not found differences in the preeclampsia rate in the order number of the insemination cycle in which pregnancy was achieved. Although this may seem to disagree with the work of Hendin *et al.* (2022), it should be pointed out that although the aforementioned authors found differences in the rate of gestational hypertension, they describe similar preeclampsia rates in relation to seminal exposure. Another study referred to a tendency to a higher preeclampsia rate in pregnancies achieved in the first cycles of artificial insemination with donor sperm (Kyrou *et al.*, 2010). This was interpreted to mean that seminal exposure might decrease the risk of preeclampsia. However, another reading could be that women predisposed to preeclampsia could become pregnant more easily. In any case, statistical significance was not reached in the aforementioned work. Given these findings of a similar rate of preeclampsia in the single women, lesbian and women with transgender partner group and heterosexual women and the absence of a relationship with the number of insemination cycles, it appears that seminal exposure does not influence the genesis of preeclampsia in our population.

The limitations of our study include all those common to retrospective studies. It also has the important limitation of dealing with data related to sexual life. For the analysis we considered two artificial groups: a) Heterosexual women with a male partner and b) Single heterosexual women, c) Lesbian women (single or in a couple) and d) Women with a transgender partner. For theoretical purposes we have assumed that the women in the single women, lesbian and women with transgender partner group had had less previous seminal exposure than those in the heterosexual group, although in no case were data available on previous seminal exposure in any of the aforementioned groups. On the other hand, the change of donor at each insemination, from the immunological point of view, could entail specific particularities.

Regarding the strengths of our study: firstly, it should be pointed out that, as far as we know, this is the first study that analyzes the influence of sexual orientation on the development of preeclampsia in artificial insemination with donor sperm. In addition, all the patients came from the same reproductive unit and were attended in the same maternity center, so the diagnostic criteria were homogeneous, with no missing cases. Specifically, the preeclampsia criterion was precise and constant, as corroborated by the different neonatal profiles of the newborns with and without preeclampsia. Moreover, this made it possible to perform a multivariate analysis to rule out possible confounding factors.

In summary, our study does not show that sexual orientation or previous sperm exposure entail an increased risk of preeclampsia, especially after correction for age and weight. Single women, lesbians and women with transgender partners can be reassured that artificial insemination with donor sperm is a safe, simple and satisfactory technique for them, as it is for heterosexual women.
